# Supplementary material for: Reconciling Mining with the Conservation of Cave Biodiversity: A Quantitative Baseline to Help Establish Conservation Priorities
Source: PLoS One. 2016 Dec 20;11(12):e0168348. doi: 10.1371/journal.pone.0168348 (PMC5173368; doi:10.1371/journal.pone.0168348)
Supplement: S1 Dataset — (ZIP) [file pone.0168348.s002.zip › Taxa/Serra Norte/SN_2013/N4WS/Lista N4WS-56.pdf]

CAVIDADE N4WS-0056							
**Classe**	**Ordem**	**Fam/Outros**	**Gên/Outros**	**Espécie**	**S-U**	**U-S**	**Zon.**
Annelida	Oligochaeta			sp.1	2	2	E/P
Arachnida	Acari	(Uropodina)		sp.1		X	E
Arachnida	Acari	Mesostigmata		jovem		X	E
Arachnida	Acari	Rhagidiidae		sp.1	X		E
Arachnida	Acari	Veigaiidae		sp.1	X		P
Arachnida	Amblypygi	Phrynidae	*Heterophrynus*	*longicornis*	1		P
Arachnida	Araneae	Filistatidae		jovem		X	E
Arachnida	Araneae	Ochyroceratidae	*Ochyrocera*	sp.1	X	X	E/P
Arachnida	Araneae	Oonopidae		jovem		X	P
Arachnida	Araneae	Pholcidae	*Leptopholcus*	sp.1		X	E
Arachnida	Araneae	Pholcidae	*Mesabolivar*	*eberhard*	X		E
Arachnida	Araneae	Salticidae		sp.3		X	E
Arachnida	Araneae	Theridiidae	*Achaearanea*	sp.1	X	X	P
Arachnida	Araneae	Theridiosomatidae	*Plato*	sp.1	X	X	P
Diplopoda	Polyxenida	Hypogexenidae		sp.1	X	X	E/P
Entognatha	Collembola	Cyphoderidae		sp.1		X	E
Entognatha	Collembola	Entomobryidae		sp.6		X	P
Entognatha	Collembola	Paronellidae		sp.1	X		P
Entognatha	Collembola	Paronellidae		sp.4	X		E
Entognatha	Diplura	Campodeidae		sp.1	X	X	E/P
Entognatha	Diplura	Projapygidae		sp.1		X	E
Insecta	Blattodea	Blaberidae		jovem	5		P
Insecta	Coleoptera	Carabidae		jovem	X		E
Insecta	Coleoptera	Dytiscidae		sp.11B	X		E
Insecta	Coleoptera	Dytiscidae		sp.12A		X	E
Insecta	Coleoptera	Elminthidae		jovem	X		E
Insecta	Coleoptera	Scarabaeidae		jovem	X		E
Insecta	Coleoptera	Staphylinidae	Pselaphinae	sp.5	X	X	E/P
Insecta	Diptera	Cecidomyiidae	Cecidomyiinae	sp.	X		E/P
Insecta	Diptera	Ceratopogonidae		jovem	X	X	E/P
Insecta	Diptera	Chironomidae		jovem	X		E
Insecta	Diptera	Culicidae		jovem	X		E
Insecta	Diptera	Dolichopodidae		sp.	X		E
Insecta	Diptera	Psychodidae	*Sciopemyia*	*sordellii*	X	X	E/P
Insecta	Diptera	Tabanidae		jovem		X	E
Insecta	Heteroptera	Reduviidae	Emesinae	jovem	1		P
Insecta	Heteroptera	Veliidae	*Paravelia*	sp.1	X		E
Insecta	Homoptera	Cercopidae		jovem		X	P
Insecta	Homoptera	Cixiidae		sp.1	X	X	E
Insecta	Hymenoptera	Formicidae	*Acromyrmex*	sp.1	X		E
Insecta	Hymenoptera	Formicidae	*Anochetus*	sp.1	X		P
Insecta	Hymenoptera	Formicidae	*Crematogaster*	sp.2	X		E
Insecta	Hymenoptera	Formicidae	*Gnamptogenys*	*striatula*	X	X	E
Insecta	Hymenoptera	Formicidae	*Solenopsis*	sp.2		X	P
Insecta	Hymenoptera	Formicidae	*Strumigenys*	sp.1		X	P
Insecta	Isoptera	Termitidae	*Atlantitermes*	sp.	X		P
Insecta	Lepidoptera	Noctuoidea		sp.1		23	E
Insecta	Neuroptera	Myrmeleontidae		jovem		1	E
Insecta	Orthoptera	Phalangopsidae	*Paraclodes*	sp.	3		E/P

Insecta	Orthoptera	Phalangopsidae	*Phalangopsis*	sp.	2	2	P
Insecta	Psocoptera	Lepidopsocidae	*Lepidopsocus*	sp.1		X	E
Insecta	Psocoptera	Psocomorpha		jovem		X	E
Turbellaria	Tricladida			sp.1	X	X	E
Amphibia	Anura	Strabomantidae	*Pristimantis*	cf. *fenestratus*	4	8	E/P
Mammalia	Chiroptera	Emballonuridae	*Peropteryx*	sp.	2	1	P
